# Cannabis use and suicidal ideation among youth: Can we democratize school policies using digital citizen science?

**DOI:** 10.1371/journal.pone.0263533

**Published:** 2022-02-14

**Authors:** Tarun Reddy Katapally

**Affiliations:** 1 Faculty of Health Sciences, Western University, London, United Kingdom; 2 Johnson Shoyama Graduate School of Public Policy, University of Regina, Regina, Saskatchewan, Canada; 3 College of Medicine, Health Science Building, University of Saskatchewan, Saskatoon, Canada; Brown University Warren Alpert Medical School, UNITED STATES

## Abstract

**Background:**

School policies and programs are important in preventing Cannabis use among youth. This study uses an innovative digital citizen science approach to determine the association between Cannabis use and suicidal ideation among youth while investigating how school health policies mediate this association.

**Methods:**

The study engaged 818 youth (aged 13–18 years) and 27 educators as citizen scientists via their own smartphones. Youths responded to time-triggered validated surveys and ecological momentary assessments to report on a complex set of health behaviours and outcomes. Similarly, educators’ reported on substance misuse and mental health school policies and programs. Multivariable logistic regression modeling and mediation analyses were employed.

**Results:**

412 youth provided data on substance misuse and suicidal ideation. Cannabis use and other factors such as bullying, other illicit drug use, and youth who identified as females or other gender were associated with increased suicidal ideation. However, school policies and programs for substance misuse prevention did not mediate the association between Cannabis use and suicidal ideation.

**Conclusions:**

In the digital age, it is critical to reimagine the role of schools in health policy interventions. Digital citizen science not only provides an opportunity to democratize school policymaking and implementation processes, but also provides a voice to vulnerable youth.

## Background

Globally, suicide is one of the most common causes of death among young people aged 10–24 years (6% of deaths) [1]. Suicidal behaviours (suicidal thoughts/ideation, suicidal attempts), which have increased among youth (13–18 years) and young adults (18–24 years), are associated with the earlier initiation of cannabis use [2]. This has led researchers to investigate if cannabis use is a risk factor that can trigger suicidal behaviours [3]. Evidence indicates that youth are especially vulnerable to mental health disorders and suicidal ideation associated with cannabis use [4]. Moreover, youth Cannabis use has been linked to an increased risk of depression and suicidal behaviour in young adulthood [5].

Numerous countries have legalized the use of cannabis for nonmedical purposes, including Canada, Georgia, South Africa, Uruguay, and some states in the United States [6]. Recent evidence in the United States showed that 16.4% of youth aged 12–17 years reported cannabis use [7]. Canada is one of the latest jurisdictions to legalize nonmedical use of cannabis, with the government of Canada legalizing the use, possession, purchase, and growth of recreational cannabis on October 17, 2018. According to the National Cannabis Survey (NCS), about 18% (5.3 million) of Canadians aged 15 or older reported using cannabis in the past three months after legalization, which indicated an overall increase in consumption (14% in 2018 Quarter 1 to 17.5% in 2019 Quarter 1) [4].

It is important to understand the mental health impacts of cannabis use among youth and young adults because evidence indicates that physiological development of the brain is not complete until entering adulthood [8–10]. One Canadian study [11] in 1043 young university students (mean age = 20 years) used questionnaires to assess perceptions of risk associated with cannabis use and its associations with anxiety symptoms. The authors observed that males were more likely to perceive minimal risk with the use of cannabis, while cannabis use was more strongly associated with anxiety among females [11].

Consistent evidence also indicates that Cannabis use is a risk factor for developing psychosis and schizophrenia among youth. A systematic review published in the year 2020, which investigated the most recent literature (2009–2019), evaluated studies on the age of initiation of Cannabis use, among other factors [12]. The systematic review showed that early initiation of Cannabis use is associated with an increased risk of psychosis by reiterating that other substance misuse should be factored in when understanding the relationship between Cannabis use and psychosis. Apart from lower age of onset of Cannabis use [13–15], potent varieties of Cannabis use [16, 17], and Cannabis use at higher frequency during adolescence [18] were associated with psychosis. Prospective investigations, such as a 15-year Swedish study among a large cohort of individuals (n = >50,000) [19], also indicate that early use of Cannabis increases the risk of schizophrenia.

The clear evidence of the association of Cannabis use and severe mental illness reiterates the importance of early interventions. Among various interventions and initiatives to address mental health and substance misuse among youth, school policies and programs offer an upstream approach to positively influence youth health behaviours and outcomes [20–23]. However, there is a dearth of evidence that takes into account school policies in understanding the relationship between cannabis use and youth mental health. In particular existing methods do not provide youth or educators a voice to inform and influence school policies [24].

This study uses data from a digital epidemiological and citizen science platform [25] that engaged youth and educators using their own smartphones immediately after the legalization of cannabis in Canada. The study determines not only the association between cannabis use and suicidal ideation among youth, but also the mediation of this association by school mental health and substance misuse policies to explore the potential of digital citizen science to democratize policy and enable youth equity.

## Materials and methods

### Design

As part of a digital epidemiological and citizen science platform (Smart Platform), [25] a quasi-experimental study called Smart Youth was conducted immediately after the legalization of cannabis in Canada [26] 818 youth (aged 13–18 years) and 27 educators in 5 out of 12 high schools in the provincial capital city of Regina, Saskatchewan, Canada, were engaged as citizen scientists via their own Smartphones. Regina is the second-largest city in the province of Saskatchewan, with a population of 215,106 according to the 2016 Statistics Canada Census [27]. All youth and educators used a custom-built digital epidemiological Smartphone application (app), specifically adapted for the study, which operates on both Android and iOS platforms. Ethics approval was obtained from the Research Ethics Boards of Universities of Regina and Saskatchewan through a synchronized review protocol (REB # 2017–29).

Smart Youth study was embedded in all participating schools’ curricula in collaboration with key stakeholders: Saskatchewan Ministries of Health, Education, and Sport. Smart Youth is informed by a Youth Citizen Scientist Council, which consists of youth from Saskatchewan. The Council engages with the research team to inform the study implementation and knowledge translation efforts.

### Recruitment

Twelve high schools in the city of Regina were approached to participate in the study, out of which 5 schools agreed to participate. One week before recruitment, schools shared implied informed consent forms with parents via email. Inclusion criteria deemed that only those youth whose parents did not object to their participation, and youth who confirmed that they did not have any medical condition that prevented them from being physically active will be recruited. Parents were given the option to reach out to the research team to opt their children out of the study.

The Smart Platform research team scheduled school visits in close coordination with schools. During each school visit, the research team conducted separate recruitment presentations to students in each grade (grades 8–12) within a school. Before these presentations were delivered, youth and educators viewed the Smart Platform’s crowdsourcing video [28] that enables the engagement of citizen scientists across the world. After class presentations, youth and educators who decided to participate in the study downloaded the custom-built app onto their own Smartphones. Both youth and educators provided informed consent via the app. The overall participation rate of youth was over 80% across all schools, thus resulting in a representative sample.

**Fig 1:**
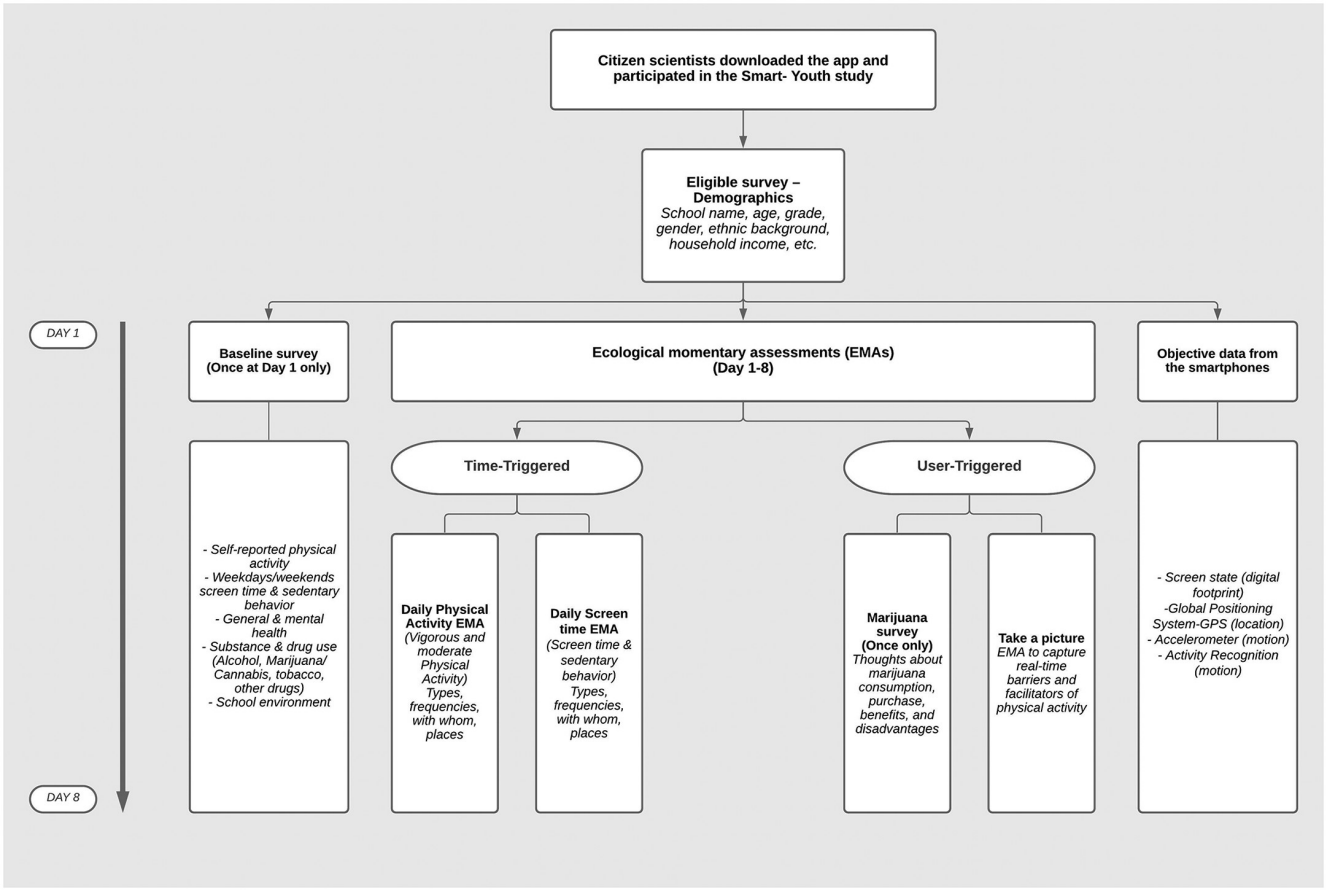
Smart youth survey deployment.

As soon as youth and educators joined the study via the app, eligibility, and baseline surveys were triggered (Smart Youth and Educator Surveys), which were completed in the presence of our team on day 1 of the study. The Smart Youth Survey (Fig 1) utilized a combination of validated questionnaires to capture a complex set of health behaviours and outcomes, including physical activity, sedentary behaviour, sleep, mental health (including anxiety and depression symptoms), and addictions.

**Fig 2:**
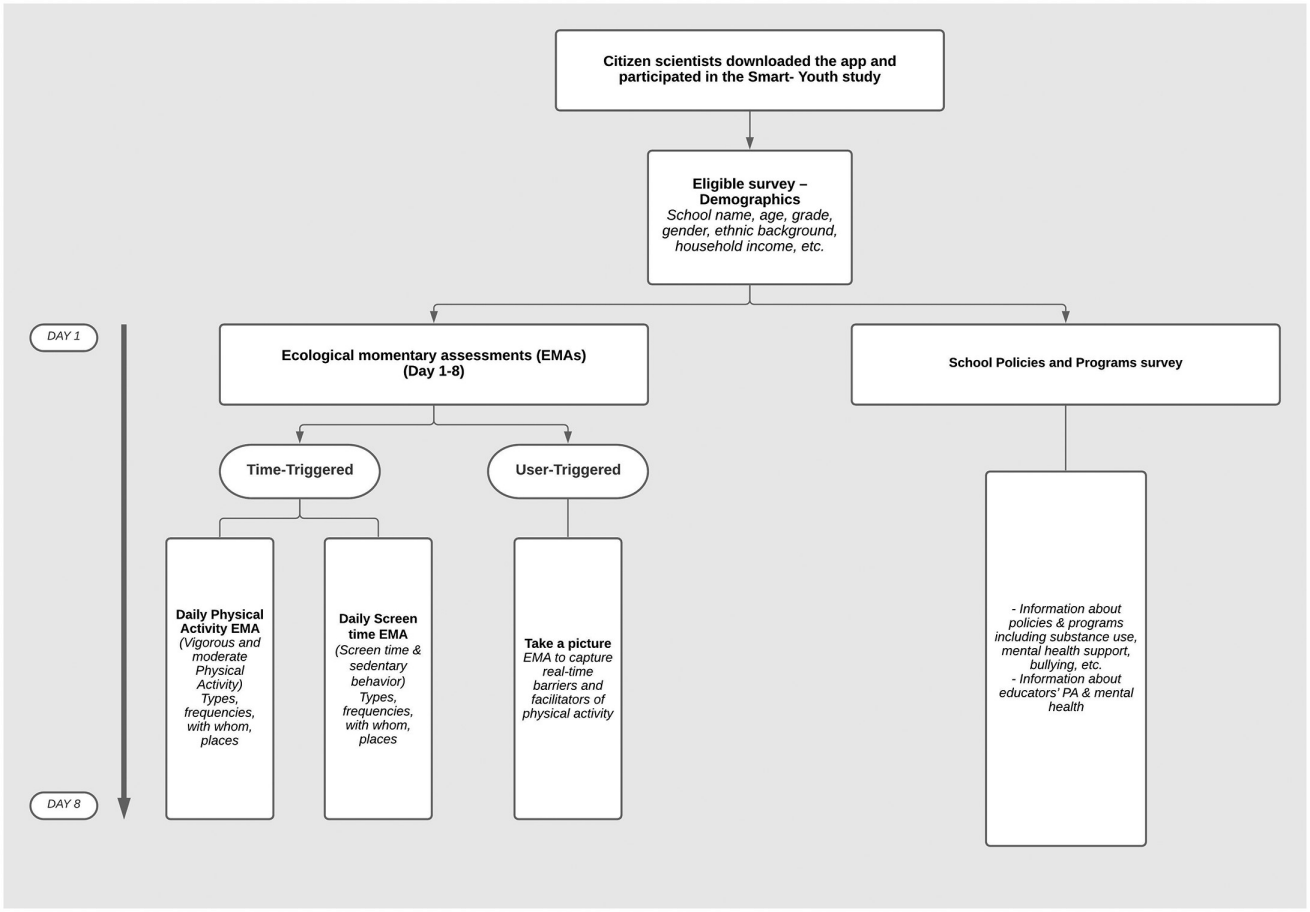
Smart educator survey deployment.

The Smart Educator Survey (Fig 2) was comprised of educators’ input regarding school policies and programs, and perceptions of how youth health outcomes could be improved. Thereafter, for the next 7 days, youth and educator citizen scientists engaged with the Smart Platform team via time-triggered ecological momentary assessments to report their perceptions on health behaviours and outcomes in real-time [29, 30].

### Measures

**Fig 3:**
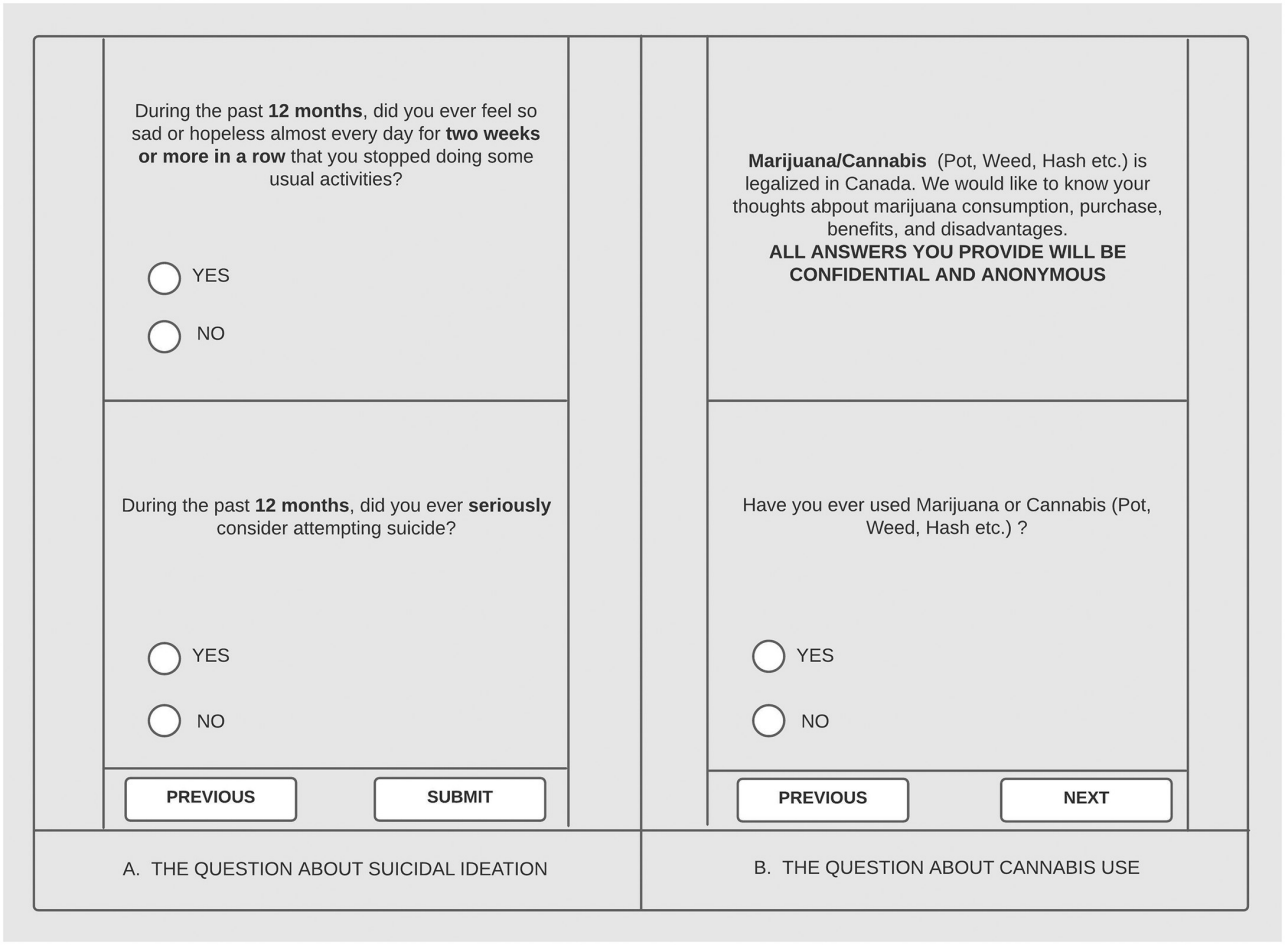
Screenshot replicas of suicidal ideation and cannabis questions.

The primary outcome of interest is *suicidal ideation* measured using the following question: “During the last 12 months, did you ever seriously consider attempting suicide? (Yes/No)” This question has been used previously in the Global School-based Student Health Survey, which was developed by the World Health Organization in collaboration with the Centers for Disease Control [31]. The primary independent variable is *cannabis use* measured by the question: “Have you ever used Marijuana or Cannabis (Pot, Weed, Hash, etc.)” (Yes/No). This question has been used in multiple surveys in Canada [32]. Screenshot replicas of these two questions as they appeared on the app were shown in Fig 3A & 3B.

Covariates included to reduce potential confounding are as follows: *Gender* (male, female, and others, including Transgender individuals); Grades (Grade 9, 10, 11, 12); *Tobacco use* (Have you ever used any tobacco related products?—Yes/No); *Alcohol use* (Have you ever consumed alcohol in your life?—Yes/No); *Other illicit drug use* (Have you ever used any of the following drugs: Cocaine, Methamphetamines, Ectasy, hallucinogenic, synthetic marijuana, steroid, prescription pain medicine without a doctor’s prescription or differently than how a doctor told you to use it—Yes/No); *bullying at schools* (-Yes/No) (Any affirmative responses to any questions related to bullying at schools such as being shoved/hit/ made fun of or teased by other students in a hurtful way, etc.); *household income* (less than $70000, $70,000-80000k, more than $80,000; *ethnicity* (Canadian, First Nations, and Other [Asian, South Asian etc.])); *parental education* (Less or equal to secondary/high school vs. post-secondary education (university or college)); *age first used marijuana or Cannabis*; *frequency of using Cannabis (a joint*, *pot*, *weed*, *hash) in the last 30 days (*Once, 2–3 times, once a week, 2–3 times a week, 4–6 times a week, every day, and did not use); *youth perceptions of access to Cannabis* (difficult, easy, do not know); *youth perceptions of the legalization of nonmedical use of Cannabis* (agree, disagree, do not know).

Educator citizen scientists provided their perception of mental health and substance misuse school policy and program availability. Information regarding the availability of school policies and programs at each school was based on a consensus of educator responses within each school. Educators responded to two specific questions: “Are there any mental health supports for youth in your school?” (Yes/No); and “Does your school provide any marijuana, or drug use (cocaine, heroin, etc.) prevention programs?” (Yes/No).

### Data and risk management

To ensure confidentiality, data were encrypted before being stored on the smartphones and streamed to servers when devices established Wi-Fi connection. Any identifiable artefacts (e.g., photos) were removed or deidentified before data analysis. Permissions built into the app are restricted so that the app cannot access personally identifiable information that is present on the smartphones (e.g., contact list or network sites visited). MAC address anonymization was used to protect citizen scientists’ data based on a simple hash algorithm. Risks and privacy management options were made clear to citizen scientists while obtaining informed consent. All citizen scientists had the option to drop out of the study or pause data gathering anytime they wished via the app. Moreover, they also had the option in the settings of the app to upload data only when they had WI-FI access and/or when they were charging their phones [30]. Clear instructions were provided regarding study withdrawal within the app.

### Data analysis

Statistical analysis was conducted using IBM SPSS Statistics version 26 (IBM Corporation, Armonk, New York). Chi-square tests were applied to test the univariate association between suicidal ideation, Cannabis use and covariates. Potential risk factors, confounders, and interactive effects were determined. Variables with p-value < 0.20 and/or known risk/protective factors and/or with clinical significance in the univariate analysis were selected for the final multivariable logistic regression model. The variables retained in the final multivariable model included those that were statistically significant (i.e., p < 0.05) as well as clinically important variables. Mediation analyses were conducted to assess whether school policies and program availability mediate the association between Cannabis use and suicidal ideation. The potential mediation effect of each variable was assessed by: ([OR_full model without mediator_ − OR_full model_]/ OR_full model_)*100. For mediation to occur, a statistically significant association between the primary exposure and the outcome must be smaller and no longer statistically significant when the mediator is included. A 5% change in the association is a meaningful difference. The Hosmer Lemeshow statistic was used to assess the goodness of fit of the final multivariable logistic regression model. The Hosmer Lemeshow statistic was greater than 0.05 in our final model, which is considered a good fit. The strength of associations was presented by odds ratios (OR) and their 95% confidence intervals (CI).

## Results

818 youth (aged 13–18 years) and 27 educators from 5 schools participated as citizen scientists in this study. 412 youth provided valid data on both the primary outcome (suicidal ideation), and the primary independent variable (cannabis use) (Table 1). The mean age of the youth citizen scientists was 15.9 years (SE = 0.07), out of which, 36.9% (n = 152) were male; and 53.9% (n = 222) were female, and 5.56% (n = 22) selected “other” as an option for gender. 25.5% (n = 105) of participants reported using cannabis at least once in their lifetime. 23.06% (n = 95) of participants reported that they seriously considered attempting suicide during the last 12 months. Youth also reported other substance use, including alcohol (61.27%), tobacco (21.65%), and other illicit drugs, such as Cocaine, Methamphetamines, Ecstasy, hallucinogenics (7.32%).

**Table 1:** Characteristics of study population (n = 412).

		N*	%
Age, mean (SE)		15.9 (0.08)	
Gender	Male	152	38.38
	Female	222	56.06
	Other	22	5.56
Ethnicity	First Nations	20	5.06
	Canadian	159	40.25
	Other	216	54.68
Cannabis use	Yes	105	25.49
	No	307	74.51
Suicide ideation	Yes	95	23.06
	No	317	76.94
Alcohol use	Yes	250	61.27
	No	158	38.73
Tobacco use	Yes	89	21.65
	No	322	78.35
Other drugs use	Yes	30	7.32
	No	380	92.68
Grade	9	114	28.57
	10	83	20.8
	11	58	14.54
	12	144	36.09
Parental education	Less or equal than secondary/high school	101	27.45
	Some post-secondary (university or college) or more	267	72.55
Age first used marijuana or Cannabis? (mean, SE)		100	14.01 (0.18)
Frequency of using marijuana or Cannabis (a joint, pot, weed, hash) in the last 30 days	Once	19	18.63
	2–3 times	8	7.84
	Once a week	4	3.92
	2–3 times a week	6	5.88
	4–6 times a week	6	5.88
	Every day	16	15.69
	I did not use marijuana in the last 30 days	43	42.16
Perceptions of getting access to marijuana	Difficult	41	10.46
	Easy	214	54.59
	I do not know	137	34.95
Perceptions of the legalization of recreational marijuana	Agree	145	37.18
	Disagree	123	31.54
	I do not know	122	31.28

Table 2 shows the characteristics of youth stratified by suicidal ideation reporting (Yes/No). Females were significantly more likely to report suicidal ideation compared to males (26.6% vs.12.5%; p = 0.008). 45.5% of respondents who selected “Other” gender category reported suicidal ideation which is significantly higher than that of males (12.5%) and females (26.6%). Youth who reported cannabis use were more likely to have suicidal thoughts compared to those who did not (40.0% vs. 17.3%, respectively; p<0.001). Similar trends of suicidal ideation were observed in those who reported other substance use: alcohol (27.6% vs. 15.8%; p = 0.006); tobacco (41.6% vs. 18.0%; p<0.001); other illicit drugs (53.3% vs. 20.8%; p<0.001). Youth who experienced bullying were significantly more likely to report suicidal ideation compared to those who did not experience bullying (34.4% vs. 13.2%; p<0.001). Youth who agreed with the policy of cannabis legalization in Canada reported a higher proportion of suicidal ideation compared to those who did not agree or had a neutral opinion (33.1% vs. 19.5% vs. 16.4%, respectively; p <0.001).

**Table 2:** Suicidal ideation difference (yes/no) across different characteristics (n = 412)*.

		Self- report of Suicidal ideation			
		Yes (n = 95)		No (n = 317)	
		N	%	N	%
Gender	Male	29	12.5**	133	87.5
	Female	59	26.6	163	73.4
	Others	10	45.5	12	54.5
Grade	9	29	25.4^¥^	85	74.6
	10	27	32.5	56	67.5
	11	5	8.6	53	91.4
	12	30	20.8	114	79.2
Cannabis use	Yes	42	40.0**	63	60.0
	No	53	17.3	254	82.7
Alcohol use	Yes	69	27.6^¥^	181	72.4
	No	25	15.8	133	84.2
Other drugs use	Yes	16	53.3**	14	46.7
	No	79	20.8	301	79.2
Tobacco use	Yes	37	41.6**	52	58.4
	No	58	18.0	264	82.0
Ever bullied	Yes	66	34.4**	126	65.6
	No	29	13.2	191	86.8
Household income	Less than 70k	16	31.4	35	68.6
	70- <80k	3	11.5	23	88.5
	>=80k	15	23.8	48	76.2
Ethnicity	First Nations	5	25.0	15	75.0
	Canadian	33	20.8	126	79.2
	Others	50	23.1	166	76.9
Parental education	Less or equal than secondary/high school	27	26.7	74	73.3
	Some post-secondary (university or college) or more	52	19.5	215	80.5
Perceptions of the legalization of recreational marijuana	Agree	48	33.1**	97	66.9
	Disagree	24	19.5	99	80.5
	I do not know	20	16.4	102	83.6
Perceptions of getting access to marijuana	Difficult	7	17.1	34	82.9
	Easy	58	27.1	156	72.9
	I do not know	27	19.7	110	80.3

** p <0.001 based on chi-square tests comparing proportions of suicidal ideation difference (Yes/No) across different characteristics.

^¥^ p <0.05 based on chi-square tests comparing proportions of suicidal ideation difference (Yes/No) across different characteristics.

Among 27 educators from 5 schools who participated as citizen scientists, even though 92.6% (25/27) reported existing mental health supports for youth in their schools, a majority also reported issues with student mental health 77.8% (21/27), as well as substance use behaviours such as alcohol 70.4% (19/27), tobacco 77.8% (21/27), and other illicit drug use 70.4% (19/27).

Educator citizen scientists also reported that prevention programs were rare, with 18.5% (5/27), 33.3% (9/27), and 11.1% (3/27) reporting knowledge of alcohol, cannabis, and tobacco, prevention programs, respectively. 48.1% (13/27) of educators reported bullying to be an issue in their schools, with the main cause of concern being online bullying 37% (10/27) followed by verbal 25.9% (7/27) and physical bullying 22.2% (6/27). Nevertheless, 77.8% (21/27) of educators also reported that their schools had policies to both prevent bullying and provide support to bullying victims.

In the multivariable analyses (Table 3), youth cannabis use was associated with an increased risk of suicidal ideation (adjusted OR (aOR) = 3.09 [95%CI = 1.21–7.89]). Other illicit drug use (aOR = 5.41 [95%CI = 1.63–17.94]) and being bullied (aOR = 3.34 [95%CI = 1.63–6.43]) were also associated with an increased risk of suicidal ideation. In terms of gender, youth who identified themselves as females (aOR = 3.73 [95%CI = 1.84–7.55]) or other gender (aOR = 5.38 [95%CI = 1.40–20.73]) were associated with an increased risk of suicidal ideation in comparison with males.

**Table 3:** Univariate and multivariate logistic regression examining the associations between cannabis and suicidal ideation in youth (n = 412).

	Unadjusted OR (95% CI)	Adjusted OR (95% CI)
Cannabis use (ref.: No)	3.19 (1.95–5.21)	3.09 (1.21–7.89)
Sex (ref.: Male)		
Female	2.53 (1.43–4.46)	3.73 (1.84–7.55)
Others	5.83 (2.21–15.34)	5.38 (1.40–20.73)
Grade (ref: Grade 9)		
Grade 10	1.41 (0.75–2.63)	1.40 (0.64–3.09)
Grade 11	0.27 (0.10–0.75)	0.11 (0.02–0.50)
Grade 12	0.77 (0.43–1.38)	0.54 (0.24–1.20)
Alcohol use (ref.: No)	2.02 (1.21–3.37)	-
Tobacco use (ref.: No)	3.23 (1.94–5.38)	1.60 (0.62–4.11)
Other drugs use (ref.: No)	4.35 (2.03–9.30)	5.41 (1.63–17.94)
Being bullied (ref.: No)	3.44 (2.11–5.63)	3.34 (1.63–6.43)
Paternal education (ref.: Some post-secondary (university or college) or more)		
Less or equal than secondary/high school	0.66 (0.38–1.13)	1.31 (0.67–2.57)
Ethnicity (ref.: First Nations)		-
Canadian	0.78 (0.26–2.31)	
Others	0.90 (0.31–2.60)	
Cannabis policy (ref.: No)	0.76 (0.48–1.22)	1.34 (0.71–2.52)

In the mediation analyses, educator reporting of school policies and programs for mental health supports and substance misuse prevention did not statistically mediate the association between cannabis use and suicidal ideation among youth.

## Discussion

Using data from a digital epidemiological and citizen science platform that engaged youth and educators as citizen scientists via their own smartphones, this study aimed to determine the association between cannabis use and suicidal ideation among youth, and the mediation of this association by school public health prevention policies and programs. We found that cannabis use was associated with an increased risk of suicidal ideation among youth, with those who identified as females and other gender showing a greater association with suicidal ideation. Other factors such as other illicit drug use (Cocaine, Methamphetamines, Ecstasy, hallucinogenics) and being bullied were also associated with increased risk of suicidal ideation. School mental health supports, and cannabis use prevention policies and programs, as reported by educator citizen scientists did not mediate the association between cannabis use and suicidal ideation among youth.

The positive association between cannabis use and suicidal thoughts has been supported by previous research [5, 8, 33]. For example, a recent systematic review and meta-analysis conducted in 2019 concluded that cannabis use during adolescence is associated with subsequent suicidal ideation within adolescence and in young adulthood with the pooled OR of 1.50 (95% CI, 1.11–2.03). The National Longitudinal Survey of Children and Youth in Canada showed that among 6,788 youth aged 14–15 that substance use increased the risk of suicidal behaviors [32]. A 30-year longitudinal study of a birth cohort of 1265 children in New Zealand showed that frequent cannabis use (at least several times a week) predicts later suicidal ideation in susceptible males [34].

We also found that the use of the other illicit drugs such as Cocaine, Methamphetamines, Ecstasy, and hallucinogenics, was associated with increased the risk of suicidal ideation, a finding that is consistent with current evidence [35–37]. This suggests that cannabis use should be targeted as part of a multicomponent substance misuse prevention plan to support youth during a vulnerable phase in their life course.

Another risk factor of youth suicidal ideation in our study was bullying, which is supported by existing evidence [38–40]. Both cross-sectional and longitudinal findings indicate that there is an increased risk of suicidal ideation among youth who are bullied [37]. This is indicative of the need for school bullying policies and programs that also take into consideration the association between substance misuse and suicidal ideation [40, 41].

Gender played a significant role in youth’s association with suicidal ideation, a finding that is consistent with previous studies [40, 42]. Youth who identified themselves as females were at higher risk of having suicidal ideation than males. Perhaps more significantly, almost half the youth who identified as belonging to a gender other than male or female reported suicidal ideation. This finding was substantiated in adjusted multivariable analyses, where the risk of association with suicidal ideation among other gender category was significantly higher in comparison with male youth.

These findings corroborate emerging evidence of suicidal ideation among youth who identify as transgender [43, 44]. Gender differences in suicidal thoughts should be taken into consideration when designing school prevention and/or support policies to reduce the risk of suicidal ideation among youth. Our findings also indicate the need for consistent use of sex and gender-based data collection processes and analyses to inform population health policies [45].

School policies and programs are known to have a direct impact on youth health [46–48]. Given the long-term consequences of cannabis use in adolescence, [49] we set out to understand the influence of school policies and programs from an educator perspective. Current evidence indicates that school-based programs can be effective in preventing and reducing the use of cannabis among youth [50–52] Such programs also increase youth awareness of the consequences of cannabis use and benefits related to non-use. Although educators citizen scientists reported school policies related to mental health and substance misuse, we weren’t able to establish that these policies mediated the relationship between cannabis use and suicidal ideation among youth.

Apart from the possibility that current school policies are not adequate to address cannabis use among youth, our innovative citizen science approach points towards several implications. There is a difference between policy presence and policy implementation. Our approach differed from existing methods of obtaining policy information from a single source to understand implementation [46–48]. We engaged a randomly selected cohort of educators from across all schools to obtain their perception of school policies, which provides us an estimate of the dispersion of school policies.

This digital citizen science approach is not only a democratic way of understanding school policy from the perception of educators (i.e., better data), but also provides a pathway for democratizing policymaking—educators and youth citizen scientists influencing school policies [24]. With respect to cannabis use, it is important to not only understand the success of school policies, but also to obtain a perception of youth about cannabis access and legalization—data which are critical for the success of policy development and implementation. Citizen science provides a voice to youth, which in itself could address mental health [24].

As understanding implementation and uptake of school policies is an essential component of policy effectiveness and efficacy, digital citizen science can also facilitate the evaluation of school policies. Perhaps more importantly, digital citizen science opens up the possibility of machine learning-informed behavioural interventions that can identify risk in real-time and provide virtual care services [53, 54]. However, to ethically engage citizens it is important to provide anonymity, privacy and data sovereignty [24].

Apart from adding to the current evidence that Cannabis use among youth is associated with increased risk of suicidal ideation [5], this study reiterates that the relationship between substance misuse and mental health is complex, where use of multiple drugs and negative experiences such as bullying play a key role [55]. This indicates the need for holistic school policy development, where silos are avoided in addressing substance misuse, mental health, and bullying. However, holistic school policies should not equate to one-size-fits-all approach, as this study showed that youth who identified as females or other gender were at a much higher risk of suicidal ideation. In practical terms of providing support at the school-level, it is important to develop targeted interventions that prioritize at-risk youth.

Nevertheless, we live in a digital world, where youth are especially susceptible to negative consequences of technology and social media [56] There is only so much we can do in physical school places when youth are literally in virtual spaces for significant portions of the day. The most significant implication of this study is the digital citizen science approach, which allows real-time engagement with youth and educators to ensure consistent engagement and prospective measurement of health behaviours and outcomes. The critical element is in providing a voice to both youth and educators, which in itself can be a positive force in mitigating the risk of substance misuse and mental illness.

The digital citizen science approach also provides a pathway for obtaining longitudinal as well as qualitative data that will not only enable effective implementation of school policies, but also facilitate the evaluation of the implementation and efficacy of school policies. The engagement of youth and educators using digital citizen science approaches goes beyond participatory action as advanced machine learning and artificial intelligence algorithms can be used to address the risk of suicidal ideation in real-time [57]. With mental health being a significant challenge among youth globally [58], the risks of not innovating are far greater and schools need to leverage the technology at hand to use it for promoting school health.

This is the first cannabis use study to engage with both youth and educators as citizen scientists to obtain both individual-level and school-level data in real-time. Our digital citizen science approach provides a pathway for obtaining longitudinal as well as qualitative data that will not only enable effective implementation of school policies, but also facilitate the evaluation of the implementation and efficacy of school policies [24]. More importantly, with the risk of suicide being the highest among youth [59, 60], it is important to look beyond existing measures that are not working and explore digital citizen science approaches using big data and machine learning to address mental health in real-time [61, 62].

### Strengths and limitations

Citizen scientist perspectives obtained through smartphone reporting are prone to recall and social desirability bias. Youth may have under- or over-reported cannabis use due to peer pressure. However, we believe the self-reported use of cannabis is relatively accurate (79.9% sensitivity and 80.0% specificity), and the smartphone engagement provides anonymity that reduces misreporting [46]. Nevertheless, objective measurement of behaviours by ethically engaging citizens using their own mobile devices would provide an opportunity to validate these findings [30, 63].

The exposures and outcomes that we examined were cross-sectional, raising issues of temporality and reverse causality. However, the direction of causality between mental health problems and cannabis use remains unclear, and our findings were supported by literature [64]. Our study does not take into account youth who may have dropped out of school, and these data should be collected in future studies. Although we engaged youth to obtain comprehensive information, residual confounders may not have been accounted for in our analysis (e.g., information about peer pressure)–another area of focus for future data collection. The lack of qualitative data about the process of school policy is a limitation that reduces our ability to understand the impact of school policies. Another key aspect that is usually not measured is the variation of behaviours across seasons in children and youth, an important factor that needs to accounted in future studies [65, 66].

Finally, Internet inequity could be an issue in obtaining a representative sample from participants, and potentially widen the gap of existing inequities [24]. Nevertheless, the primary strength of our study is that it provides insight into both big data collection and policy interventions using digital citizen science. With over 3 billion smartphones in global usage currently [67], the ability to engage as citizen scientists change the landscape of population health research [68] In fact, all youth and educators who agreed to participate in our study owned smartphones with data plans. As part of the digital citizen science-based Smart Platform [28], to address potential Internet inequity, we work with schools to ensure that all youth and educators who participate as citizen scientists receive access to mobile phones and data plans [24].

## Conclusions

Irrespective of the variation of cannabis legalization across global jurisdictions, cannabis access and use among youth is an important public health issue. With the risk of suicide being very high among youth, and with cannabis use being associated with the risk of suicidal ideation, it is critical to reimagine the role of schools in health policy interventions. Digital citizen science not only provides an opportunity to democratize school policymaking and implementation processes, but also provides a voice to vulnerable youth, which in itself could improve mental health and facilitate equity in the 21^st^ century.
