# Correction: Cannabis use and suicidal ideation among youth: Can we democratize school policies using digital citizen science?

**DOI:** 10.1371/journal.pone.0293934

**Published:** 2023-11-01

**Authors:** Tarun Reddy Katapally

There is an error in affiliation 1 for author Tarun Reddy Katapally. The correct affiliation 1 is: Faculty of Health Sciences, Western University, 1151 Richmond St, London, ON N6A 5B9.
